# Stress echocardiography: safety and tolerability

**DOI:** 10.1186/1476-7120-11-30

**Published:** 2013-08-20

**Authors:** Nada Fennich, Fedoua Ellouali, Salima Abdelali, Assad Chaara, Allal Berrada, Imane Elhajjaji, Rhizlane Cherradi, Saadia Abir, Nawal Doghmi, Mohammed Cherti

**Affiliations:** 1 Cardiology B Department, Ibn Sina University Hospital, Secteur 16 bloc I num 8, Hay Riad, Rabat, Morocco; 2 Cardiology Department, Agdal Clinic, Rabat, Morocco; 3 Laboratory of Biostatistics, Clinical Research and Epidemiology, Faculty of medicine and pharmacy, University Mohammed V Souissi, Rabat, Morocco

**Keywords:** Stress echocardiography, Dobutamine, Exercise, Safety

## Abstract

**Background:**

Stress echocardiography is a valuable tool for the noninvasive diagnosis of ischemic heart disease. Despite its widely use in the clinical practice, safety and side effects profile have never been evaluated in Moroccans.

**The aim:**

To assess the safety and tolerability of the two stress echo modalities in Moroccans.

**Methods:**

The study was made by 311 patients with known or suspected coronary artery disease, 203 underwent exercise echocardiography and 108 underwent dobutamine echocardiography, major and minor rhythmic complications and side effects were recorded for the two groups.

**Results:**

We registered 3 (2, 8%) major rhythmic events in the dobutamine group (2 sustained supraventricular tachycardia and 1 sustained ventricular tachycardia), there was no major rhythmic events in the exercise group. Minor rhythmic events were frequent (43, 5% in the dobutamine group and 19, 2% in the exercise group with a p = 0, 0001). Severe hypotension occurs in 4 (3, 7%) patients during a dobutamine stress, there was no significant drop in the blood pressure during exercise stress procedures. Non cardiac side effects were more common among patients who underwent a dobutamine stress echo (13, 9% vs. 3, 4% with p = 0,001).

**Conclusion:**

Exercise is safer than dobutamine stress echocardiography, complications and adverse effects with the use of dobutamine are usually minor and self-limiting.

## Introduction

Stress echocardiography is an established clinical testing method for the detection of coronary artery disease. The exam end point is the development of a left ventricular regional myocardial dysfunction traducing a transient imbalance between oxygen demand and supply in ischemic conditions. The myocardial wall motion abnormality is an earlier and a more sensitive marker for myocardial ischemia than electrocardiographic changes or chest pain alone.

Exercise used for the first time in combination with a two dimensional echocardiography in 1979, is a simple and physiologic tool. Exercise stress echocardiography should be the first choice in patients with suspected coronary artery disease. Pharmacological stressors have been developed to evaluate patients unable to exercise with neurological, orthopedic, peripheral vascular or respiratory problems, dobutamine is the most used.

Safety and tolerability of the different stress modalities have been investigated in numerous studies. This is the first study conducted in Moroccan labs.

## Methods

### Population study

We retrospectively studied 311 patients with known or suspected coronary artery disease, referred to our department for a myocardial ischemia assessment between 2006 and 2012. 203 underwent exercise stress echocardiography (160 males, mean age 59.2+/−8.6 years) and 108 had dobutamine stress echocardiography (48 males, mean age 62.9+/−11.3 years). Clinical characteristics including a history of diabetes, hypertension, hypercholesterolemia, cigarette smoking, ischemic disease related medications, prior myocardial infarction, prior percutaneous coronary angioplasty and prior coronary artery bypass grafting were recorded for all patients.

### Stress protocol

Exercise stress echo was conducted using a semi-supine bicycle ergometer with 25 W incremental loading every 2 minutes. At the end of each stage, the heart rate, the blood pressure and a 12-lead ECG were recorded. Echocardiographic images were acquired with the patient on the bicycle at rest, at peak exercise and at recovery period.

Pharmacological stress echo was performed using dobutamine. Dobutamine was administered intravenously with an infusion pump, beginning at a dose of 5 to 10 μg/kg per minute and increased by 10 μg/kg every 3 minutes up to a maximum of 40 μg/kg per minute. Atropine was added at doses of 0.25 mg each minute to a maximum of 2 mg when starting the dobutamine dose of 20 μg/kg/min if heart rate was <100 beats/min. The blood pressure, the heart rate and clinical symptoms were monitored; a 12-lead electrocardiogram was obtained at baseline, at the start of the dobutamine infusion and at the end of each 3-min interval. Echocardiographic images were acquired at rest, at small doses, at peak dose and at recovery period.

### Echocardiographic analysis

Images in standard views were acquired and displayed side by side in a quad-screen format. All images were recorded on videotape and digitized in continuous-loop format. The left ventricle was divided into 17 segments according to the recommendations of the American Society of Echocardiography [1]. A 4-point score was assigned to each segment as follows: 1 = normal, 2 = hypokinesia, 3 = akinesia, and 4 = dyskinesia. A wall motion score index was derived by dividing the sum of individual segment scores by the number of interpretable segments. A normal stress response is defined by a uniform increase in wall motion and systolic wall thickening, with a reduction in end-systolic cavity area. Ischemic response was defined as the development of new or the worsening of pre-existing wall motion abnormalities in 2 contiguous segments. The “biphasic” response (low-dose improvement followed by high-dose deterioration) was also regarded as criterion for ischemia [2], whereas rest akinesia becoming dyskinesia was not. Images analysis was made by the same experimented operator (N.D).

### Tests interruption

Stress echo was stopped when 85% of age-predicted target heart rate was reached, if the patient developed severe chest pain, ST segment elevation > 0.1 mV at 80 ms from the J point, new segmental wall motion abnormalities or significant adverse effects.

### Adverse events definitions

Major rhythmic events are defined as a life threatens rhythmic complications (cardiac asystole, advanced atrioventricular block, ventricular fibrillation or ventricular sustained tachycardia) or complications that require hospital admission (supraventricular tachycardia).

Minor rhythmic events are defined as the development of uniform or multiform premature ventricular beats, ventricular bigeminy or couplets and nonsustained ventricular tachycardia.

Severe hypotension was defined by an arterial pressure drop ≥ 40 mmHg with symptoms.

Minor side effects were defined as the development of headache, nausea and muscular pain.

### Statistical analysis

Continuous variables are expressed as means ± standard deviations and categorical variables are expressed as percentages. To compare between the dobutamine echocardiography group and the exercise echocardiography group, the continuous variables were analyzed using Student’s t-test and the categorical variables were analyzed using the Chi-square test. A p < 0.05 was considered statistically significant.

The statistical analysis was performed using Statistical Package for the Social Sciences (SPSS) version 18.0 (SPSS Inc., Chicago, IL, USA).

## Results

A total of 311 patients were enrolled, the average age was 61 years old, and 67% were male. The study population comprised two groups: The exercise group which included 203 patients and the dobutamine group which included 108 patients. Demographic and clinical characteristics of the two groups are represented in Table 1.

**Table 1:** Demographic and clinical characteristics of the population study

**Demographic and clinical characteristics**	**Stress modality**		**P**
	**Exercise**	**Dobutamine**	
Age (years)	59.2	62.9	0.001
Masculine gender (%)	78.9	44.4	0.0001
Diabetes (%)	27.1	44.4	0.002
Hypertension (%)	38.4	59.3	0.001
Hypercholesterolemia (%)	35	35.2	NS
Smoking (%)	47.8	26.9	0.0001
Optimal medical treatment (%)	53.4	41.7	NS
Prior myocardial infarction (%)	21.07	11.11	0.029
Prior coronary angioplasty (%)	53.7	20.4	0.0001
Prior coronary artery bypass grafting (%)	1	6.5	0.01
Left ventricular dysfunction (%)	13.79	11.11	NS

*NS* Non-significant.

Stress tests were maximal in 81.37% for exercise echo and 88.34% for dobutamine echo (p = NS); the percentage of age predicted heart rate reached by exercise was 90.51% and 92.89% for dobutamine. The maximal heart rate was 145.42+/−17.29 beat per minute for exercise and 146.00+/−18.17 beat per minute for dobutamine with a non-significant difference. Exercise was associated with higher maximal systolic blood pressure (190.25+/−29.03 mmHg versus 170.73+/−35.67 mmHg, p = 0.0001) and higher maximal diastolic blood pressure (91.37+/−16.18 mmHg versus 80.50+/−16.58 mmHg, p = 0.0001).

Echocardiographic positive responses were more frequently observed with the dobutamine echocardiography. Results of the two stress tests are represented in the Table 2.

**Table 2:** Results of the stress tests in the study population

**Results**	**Stress modality**		**P**
	**Exercise**	**Dobutamine**	
Echocardiographic positivity (%)	13.2	23.1	**0.003**
Major rhythmic events (%)	0	2.8	NS
Minor rhythmic events (%)	19.2	43.5	**0.0001**
Severe hypotension (%)	0	3.7	NS
Minor side effects (%)	3.4	13.9	**0.001**

*NS* Non significant.

In the presented study, no death, cardiac rupture or myocardial infarction were registered.

**Figure 1:**
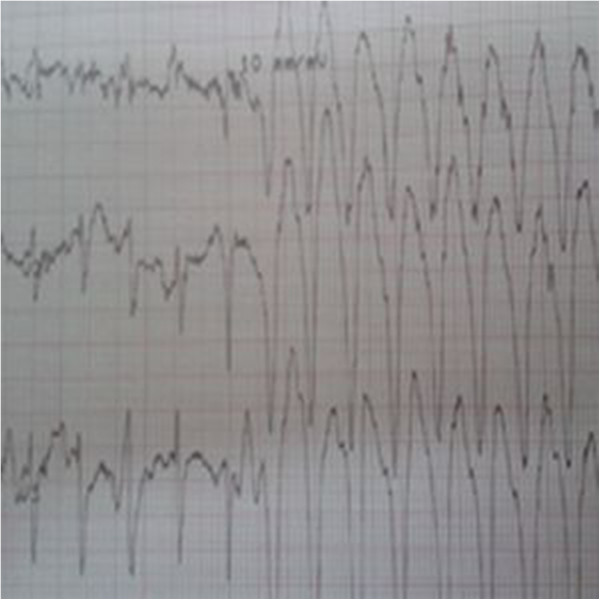
Electrocardiogram showing a run of ventricular tachycardia.

Dobutamine infusion showed higher incidence of rhythmic complications, major events were observed in 3 patients (2 sustained supraventricular tachycardia and 1 sustained ventricular tachycardia (Figure 1)). Supra ventricular tachycardia was registered in 2 patients (a 70 year-old women and a 55 year-old man) with no history of arrhythmia, with a normal left ventricular function and no induced myocardial ischemia. Ventricular tachycardia occurs in a 65 year-old diabetic patient with no history of myocardial infarction, addressed for silent myocardial ischemia screening, the left ventricular function was normal, the dobutamine stress echo was negative. In the exercise group, there was no major rhythmic event.

Minor rhythmic complications were registered in 19.2% in the exercise group and in 43.5% in the dobutamine group. In the exercise group, minor events included isolated supra ventricular premature beats in 3 patients (1.47%), isolated ventricular premature beats in 31 (15.27%), bigeminy or couplets in 4 (1.97%) and non-sustained ventricular tachycardia in 1 (0.49%). In the dobutamine group isolated supra ventricular premature beats occurs in 5 patients (4.62%), isolated ventricular premature beats in 34 (31.48%), bigeminy or couplets in 7 (6.48%) and non-sustained ventricular tachycardia in 1 patient (0.92%). All minor rhythmic complications were well tolerated.

No blood pressure decrease was seen during exercise but dobutamine infusion provoked severe systolic pressure fall in 4 (3.7%) subjects. 1 developed convulsions while 3 had only vertigo. Myocardial ischemia was present in 2 patients.

Non cardiac side effects appeared with both physical exercise and dobutamine infusion with higher incidence with the use of dobutamine as stressor.

In univariate analysis, dobutamine infusion provoked more minor rhythmic complications and non-cardiac side effects than the physical exercise.

Dobutamine infusion was associated to atropine administration in 78.5% of dobutamine tests, the mean dose was 0.66+/−0.55 mg. Adverse events were comparable in the group of patients with and without atropine co administration: major rhythmic complications occur in 3.6% versus 0 (p = NS), minor rhythmic complications were registered in 41.7% versus 52.2% (p = NS), hypotension was observed in 4.8% versus 0 (p = NS) and non-cardiac side effects appeared in 15.47% versus 8.7% (p = NS).

The use of atropine in dobutamine tests was not associated to higher incidence of adverse events.

## Discussion

Both stress modalities are safe. The occurrence of life threatening complications is rare. Data from the International Stress Echo Complication Registry reports a rate of 1 of 6574 patients undergoing exercise stress Echo and 1 of 557 patients undergoing dobutamine stress Echo [3].

In our study no death, myocardial infarction, ventricular fibrillation or high conduction disturbances were observed. Several studies reported an incidence of 0.002%, 0.02%, 0.04% and 0.23% respectively with the use of dobutamine as stressor [4]. We registered 1 case of sustained ventricular tachycardia in the dobutamine group, the reported incidence is 0.15% [4]. The clinical significance of Dobutamine stress echo induced ventricular tachycardia has not been clarified. Previous studies [5-7] failed to establish a relationship between this ventricular rhythm disturbance and inductile myocardial ischemia. Elhendy et al. [8], in a series of 286 patients who underwent dobutamine stress echo and subsequent coronary angiography, reported that tachyarrhythmia during dobutamine stress were not predicted by the presence or the extent of coronary artery disease on angiography nor by the induction of ischemia during the stress echo. The arrhythmia may be attributed to beta 1-receptor stimulation, to dobutamine induced reduction in ventricular refractory period [9] or to a dobutamine-induced reduction in plasma potassium [10].

In the presented study, supraventricular tachycardia occurred in 2 (1.8%) dobutamine tests. Similar results were reported by Secknus et al. (1.7%), Pezzano et al. (1.6%) and Tsutsui et al. (1.6%) [11-13]. Supraventricular arrhythmias seem to be more frequent in older patients [14].

Minor rhythmic complications were observed in both stress modalities with a statistically higher incidence in the dobutamine group. Minor events included premature ventricular beats and nonsustained ventricular tachycardia NSVT, the reported incidence is 33.7% and 2.1% respectively [12]. In a previous study, the prognostic significance of NSVT during dobutamine stress echo was explored, there was no difference in survival over the 3 years follow-up between the NSTV and the no NSTV groups in patients without inducible ischemia and with an ejection fraction > 0.45 [15].

Severe hypotension occurred in 3.7% of dobutamine tests, the reported incidence is 20% [4]. Although hypotensive response during exercise has strongly been associated to myocardial ischemia and poor cardiac prognostic, hypotension during dobutamine infusion cannot be consider as a specific indicator of cardiac anomalies [16]. The mechanism of hypotensive response during dobutamine infusion remains unclear, vigorous myocardial contraction around a small chamber may trigger sympathoinhibition and increased parasympathetic discharge, leading to a systemic hypotension [17]. The prognostic significance has been widely discussed, most studies did not reported a significant association with cardiac complications occurrence, while Dunkelgrun et al. in a retrospective study of 3381 patients showed that severe hypotension during dobutamine infusion is an independent predictor of cardiac death and non-fatal myocardial infarction [18].

Atropine co administration in dobutamine stress echocardiography is a safe and effective strategy in patients who had inadequate chronotropic response. Atropine adjunction was first reported by Mc Neill et al. [19], if 85% of maximal predicted heart rate was not achieved at the end of the last stage, atropine was administered in doses of 0.25 mg each minute to a maximum of 1.0 mg while maintaining a continuous infusion of dobutamine. Recently, new protocols have been developed including the early injection of atropine at the dose of 20 μ/Kg/min when the heart rate is < 100 beats per minute. The early injection of atropine during dobutamine stress echo has been demonstrated to reduce the duration and dose of dobutamine infusion, to reduce dobutamine related adverse events, while at the same time preserving a similar diagnostic accuracy.

In our study, we used the early atropine protocol. Atropine administration was not associated to higher incidence of adverse events [20].

Stress modalities are safe but not equally safe, exercise tests are safer. The most common adverse events are the rhythmic disturbances and are usually minor and well tolerated.

## Competing interests

The authors declare that they have no competing interests.

## Authors’ contributions

Concept and design: FE, ND. Supervision: RC, AB. Resources and Material: SA, AC. Data collection and processing: NF. Analysis and interpretation: FE. Literature search: NF, IE. Writing: NF, FE. Critical review: MC. All authors read and approved the final manuscript.
